# Fundamental Motor Skills Intervention for Children with Autism Spectrum Disorder: A 10-Year Narrative Review

**DOI:** 10.3390/children7110250

**Published:** 2020-11-23

**Authors:** Silvia Busti Ceccarelli, Camilla Ferrante, Erica Gazzola, Gian Marco Marzocchi, Maria Nobile, Massimo Molteni, Alessandro Crippa

**Affiliations:** 1 Child Psychopathology Unit, Scientific Institute, IRCCS E. Medea, 23842 Bosisio Parini, Italy; silvia.busti@lanostrafamiglia.it (S.B.C.); maria.nobile@lanostrafamiglia.it (M.N.); massimo.molteni@lanostrafamiglia.it (M.M.); 2 Department of Psychology, University of Milano-Bicocca, 20126 Milan, Italy; c.ferrante1@campus.unimib.it (C.F.); e.gazzola1@campus.unimib.it (E.G.); gianmarco.marzocchi@unimib.it (G.M.M.)

**Keywords:** autism spectrum disorder, fundamental motor skills, intervention, treatment, rehabilitation

## Abstract

In the past decade, converging evidence has suggested that motor impairment is one of the most consistent markers, alongside sociocommunicative difficulties, for autism spectrum disorder (ASD). Indeed, widespread anomalies of movement have been described in the ASD context. These motor abnormalities could have critical implications for subsequent cognitive and social development. Nevertheless, this area of development is particularly underexamined in the autism-related context, and early intervention programs commonly focus on the core symptoms of the condition. In the present work, we review and discuss the findings from recent studies that investigated the effect of interventions regarding fundamental motor skills in autistic children. Although the limited nature of the literature prevents researchers from drawing definitive conclusions, the results from the studies discussed here demonstrated potentially significant improvements in the motor abilities of autistic children after the interventions. Only a subset of the reviewed studies explored possible changes in the sociocommunicative domain after the motor skills improvements, and they had not concordant, although promising, conclusions. Overall, in consideration of the well-documented motor impairment people with the condition, the present findings highlight the importance of including motor skills training within the rehabilitation programs designed for autistic children. Furthermore, this narrative review encourages future interventional trials to consider motor skills as a possible target for reducing activity limitations and participation restrictions of autistic children.

## 1. Introduction

Autism spectrum disorder (ASD) is a pervasive neurodevelopmental condition that is characterized by an impairment in social interaction and communicative skills, as well as the presence of repetitive and restricted behaviors. Although not included in the Diagnostic and Statistical Manual of Mental Disorders-Fifth Edition (DSM-5) criteria [1], ASD is frequently associated with extensive motor abnormalities (see [2], for a review). From the comprehensive meta-analysis of Fournier and colleagues [3] that suggested a remarkable effect size of 1.20 for motor deficits, the study of motor function in ASD has gained increasing momentum over the last decade. Indeed, a very recent analysis of the SPARK (Simons Foundation Powering Autism Research for Knowledge) study database [4] indicated that 86.9% of autistic children/adolescents are also at risk of a developmental coordination disorder. Motor difficulties in ASD occur in the first years of life, even preceding the social-communication deficits, and tend to increase with age, reducing the possibility of social interaction [5,6]. A number of studies also suggested that motor difficulties could be one of the earliest identifiable manifestations of ASD in clinical settings [7,8,9,10,11,12]. Early motor disturbances could even provide crucial information for delineating the different trajectories for typical development as well as early-onset and regressive forms of ASD that start from six months of age [13,14,15].

In this framework, the term “fundamental motor skills” (FMS) is often used to indicate the essential movements that allow a person to successfully perform a variety of physical activities—such as walking, running, jumping, reaching, catching, and throwing [16]. The development of FMS has been associated with active play throughout the childhood [17]. The stage of rhythmic stereotypies in infancy, for example, indicates neuromuscular maturation and promotes the subsequent control of specific motor patterns, whereas rough-and-tumble behavior during childhood serves as a basis for social interaction [17]. Poor motor skills represent a barrier to participation in physical activities, and the difficulty in engaging in active play prevents favorable occasions for the development of motor functions. Accordingly, motor delays in autistic children not only impact the motor domain but also may critically interfere with a range of abilities—such as cooperation, empathy, joint attention, self-regulation, and emotional well-being—that children with typical development acquire during free active play with parents, siblings, or other peers [6]. Conversely, it is important to bear in mind that also social functioning influences physical activity (even though to a lesser extent), as recently indicated by Reinders and colleagues [18]. For autistic people, social difficulties may indeed represent barriers to physical activity [18]. Although Esposito and Pasca [19] proposed that motor symptoms are an early biomarker of ASD and Leary and Hill [20] suggested that motor control is crucial for communication and social interaction, there is a lack of literature addressing this topic at this stage. Rehabilitation programs usually target social interaction, communicative skills, and behavioral difficulties as their primary foci. However, as discussed above, improving motor skills in autistic children could have a beneficial cascade effect on engagement in active play, thus offering remarkable opportunities for the social interactions mediated by physical activity [21,22,23].

Researchers [24] have proposed different strategies for interventions that help children cope with motor impairments in ASD. Most of the interventions involve FMS only marginally and instead focus on physical aerobic exercise, educational games, and sports, with moderately positive benefits reported in almost all studies (for reviews on this topic, see [6,25,26], for meta-analyses [24,27]). Interestingly, one systematic review [28] and one meta-analysis [29] have been performed in the last year focusing on the effect of specific interventions on FMS and gross motor skills. While reporting a positive effect of motor interventions on FMS and gross motor skills, both works provided useful insights to start developing a specific guideline for building future interventional studies. Namely, Colombo-Dagouvito and Block [28] very precisely defined a motor intervention as “an intentional and directed manipulation of motor skills through a set procedure taking place over a defined period of time to develop an overall change in motor skill performance” (p. 162). On the other hand, Case and Yun [29] clearly demonstrated that interventions that were 16 total hours or longer had a significantly larger effect than those shorter than 16 h. Furthermore, the authors also indicated that interventions in experimental settings were more effective than ones in practical settings [29].

Given the current stage of literature and starting from these recent suggestions, the primary goal of the present study was to summarize the studies on FMS interventions in autistic children in the past 10 years to delineate the strengths and weaknesses of the previous programs and to continue encouraging future research in this field. Being exploratory in nature, this work did not plan to exhaustively review all interventional studies addressing FMS as recently done [28,29]. Our focus was rather to discuss the most recent evidence also in light of the advances in autism-related research in the last 10 years. A secondary objective of this narrative review was to investigate whether interventions that have specifically addressed FMS included measures related to autistic core features and, in case, to explore the possible effect of FMS interventions on the sociocommunicative domain of autistic children. Given the above-mentioned literature [18], it would be plausible to expect some improvements in the social domain after FMS motor intervention. Moreover, exploring the potential effect of FMS programs beyond the motor domain is particularly relevant when considering the International Classification of Functioning, Disability, and Health for Children and Youth (ICF-CY; [30]) perspective. Indeed, ICF-CY assumes that the degree of functioning of a child is the result of a complex interaction between body functions and structures, activities, and participation, and that changes in one of these levels may influence the others. Accordingly, significant modifications of body functions (i.e., motor skills) after FMS intervention could significantly affect also “interpersonal interactions and relationships” within the level of activity and participation, a component significantly impaired in ASD [31].

## 2. Methods

A comprehensive search of three databases (PubMed, PsychInfo, Web Of Science) from January 2010 up to September 2020 was performed by two reviewers (S.B.C. and E.G.) using a combination of indexing terms with free text searching. The form of the general search strategy was the following: ([motor] AND [intervention] AND [autism] OR [autism spectrum disorders]). In the present review, studies were included if they involved motor intervention that fulfilled the definition provided by Colombo-Dagouvito and Block [28] and that specifically addressed FMS. Other inclusion criteria were (a) participants were 3–12 years old and (b) specific fine and/or gross motor intervention. Exclusion criteria were: (a) participants were above 12 years old; (b) general sport and physical activity intervention; (c) rehabilitation treatment or programs not specifically developed to involve motor intervention targets (e.g., Early Start Denver Model, JASPER); (d) single case studies. Despite a number of advantages, single case studies were not included because of the lack of generality of obtained effects.

**Figure 1:**
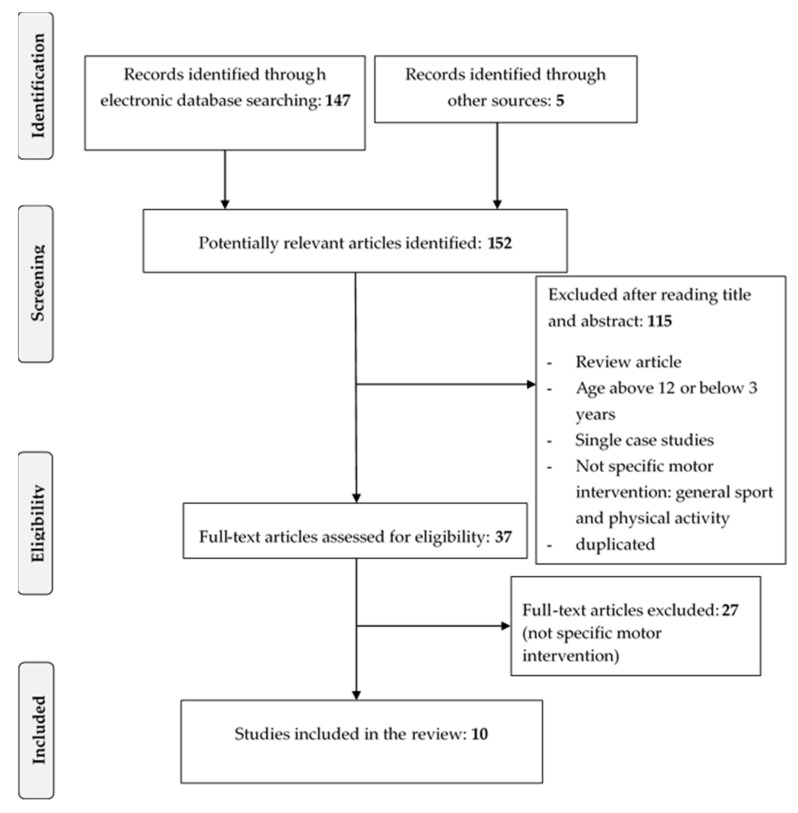
Flow diagram of the study selection process.

The search and inclusion/exclusion criteria were specified before conducting the review but were not registered online. The search process through electronic databases identified a total of 147 studies. The electronic search was supplemented by hand searches of journals and individual article reference lists. Five additional articles were identified. Of the total 152 studies, 115 were excluded after reading the title and the abstract. The two reviewers then carried out a full-text screening of the remaining 37 articles. A total of ten articles were identified that met all inclusion/exclusion criteria and were included for analysis. Figure 1 depicts a flow diagram showing the procedure for the present review and the number of articles examined.

The included studies are summarized in Table 1.

## 3. Results

### 3.1. Participants: Clinical and Demographic Characteristics

All the participants of the studies included had a diagnosis of either autism or pervasive developmental disorder not otherwise specified, or ASD, except one case that was included in the sample due to his ASD-like characteristics [6]. Diagnoses were confirmed by a family physician, pediatrician, psychiatrist, psychologist, school psychologist or psychological associate according to DSM-IV or DSM-5 criteria in 80% of studies. In the remaining studies, the diagnostic assessment was done by two graduate-level students [38] and by the rehabilitation campus staff [34], respectively.

The age range of the participants in the reviewed studies was 3–12 years. The mean age of the studies included was seven years. The majority of the participants were male (80%).

### 3.2. Studies Designs

With respect to the experimental designs, 80% of studies were pre-/posttest quasi-experimental or experimental design. Two studies were randomized clinical trials [5,34].

### 3.3. Types of the Interventions

As for the types of interventions, 70% of studies [6,32,34,35,36,37,38] proposed a program mainly based on FMS that involved teaching and strengthening locomotor and object-control skills—such as balance, running, underhand rolling, galloping, leaping, underhand throwing, jumping, dribbling and bouncing, overhand throwing, catching, hopping, kicking, and striking. Another study focused on whole-body movements [5], but it differed from the above-mentioned works because it used the innovative support of a robot in one of the two experimental conditions. The study implemented rhythmic training as one condition and robot training as another, whereas the control training group worked on activities at a table. More precisely, the rhythm intervention included the following: imitation; praxis tasks—such as action songs with finger play, discrete whole-body movements, a xylophone game; interpersonal synchrony-based joint-action games—such as beat keeping and music making; and movements such as jumping, stomping, marching, clapping, and skipping. The robot training included the same motor activities and interpersonal joint-action games; the only difference was that, instead of having the human trainer of the rhythm intervention, the robot intervention had robots that guided the participants through the activities of the sessions. Whereas the rhythm and robot conditions of the intervention focused mainly on whole-body and gross motor skills, the control condition focused on promoting academic and fine motor skills (by building creations using Play-Doh or LEGO blocks or engaging in arts and craft activities—such as drawing, coloring, and cutting) to resemble the training that autistic children typically receive in special education programs. Pfeiffer et al. [39] also used training in fine motor skills that focused on construction, drawing, writing, and crafts activities and compared it to a sensory integration treatment that was characterized by tactile, vestibular, and proprioceptive experiences and promoted enhanced sensations, adaptive responses from the child, and a good therapist–child relationship. In another innovative study, Edwards et al. [33] used active video games (AVGs) to improve actual and perceived object control in autistic children. For this intervention, the authors used the games Kinect Sports Season 1, Kinect Sports Season 2, Sports Rivals, and Kinect Adventures for Microsoft’s Kinect platform, which is associated with Xbox 360 and Xbox One.

### 3.4. Durations of the Interventions

On average, each training session lasted 45–60 min, apart from the study of Felzer-Kim and Lynn Hauck [34] that implemented sessions of 15 min each. Eighty percent of studies had an intensity of 1–3 h/week [5,6,32,33,34,36,37,39] and 60% had a total duration of 6–12 weeks [5,6,32,36,38,39]. Other two studies had higher intensity. The first one consisted in 20 h/week, five days/week [38]. The second one was defined as “five full days’ program” [35]: it was a camp curriculum inspired by Special Olympics Fundamentals program and training on motor skills was integrated in active group games in order to promote a high level of practice in more naturalistic situations. Lastly, another three studies presented durations which differed from the average: Edwards and colleagues’ study [33] lasted two weeks, whereas the studies of Felzer-Kim and Lynn Hauck [34] and of Henderson and colleagues [37] lasted 20 and 24 weeks, respectively. It is worth noting that only Bremer et al.’s study [32] had a crossover design, though the two arms had different intensities: in the first phase, the experimental group received an FMS intervention in one 1 h session per week over 12 weeks (12 sessions), whereas the control group received the same FMS intervention in 2 h sessions per week for six weeks (12 sessions) in the second phase, so that has been possible comparing groups for the efficacy of different intensities.

### 3.5. Settings

The interventions took place in different settings. Seventy percent of interventions took place at the participants’ schools [6,35,38], or at rehabilitation centers [32,34,37,39]. Two studies involved participants’ homes [5,33], and one study performed the program at a sports arena [36]. The interventions were implemented by therapists [5,34,39], researchers (either principal investigators or research assistants; [6,32,33,34,38]), teachers/sport coaches [35,37], or parents [5,33].

### 3.6. Outcome Measures and Results

Motor outcomes were assessed in the considered studies using different test of motor development: Peabody Developmental Motor Scales-2 (PDMS-2), the Test of Gross Motor Development-2 and -3 (TGMD-2, -3), and the Bruininks–Oseretsky Test of Motor Proficiency-2 (BOT-2). All the considered studies revealed statistically significant differences between the experimental and control groups or a significant within-group improvement in at least one of the outcome measures after the interventions, apart from the study of Felzer-Kim and Lynn Hauck [34], which, nevertheless, presented midpoint results of a larger, ongoing intervention. With respect to the motor domain, results presented here are organized in macroareas for the sake of simplicity. However, it is important to consider that each of the above mentioned motor assessment measures has different underlying assumptions, according to the theoretical perspective from which the test was developed [40]. The results showed significant improvements in:

(a) fine motor abilities, including fine motor precision and fine motor integration, tested by BOT-2 [36];
(b) object control, intended as developmental abilities needed to catch and throw objects as assessed with PDMS-2 [32], and grouping a set of components, such as striking a stationary ball, stationary dribble, kick, catch, overhand throw, and underhand roll as tested by TGMD-2 [6,35,37,38];
(c) locomotor skills, as measured with TGMD-2 [6,35,37,38];
(d) global gross motor skills, as quantified by the overall composite score of TGMD-2 [35], by four subtests of PDMS-2 (reflexes, stationary, locomotion, and object manipulation) [32], and by total motor composite score of BOT-2 [36]);
(e) body coordination, as assessed with BOT-2 [5].

Edwards et al. [33] observed increased self-perceptions of proficiency in object-control skills after AVG training only in the ASD group, even though neither the TGMD-3 scores nor the quantitative measures of the object-control skills showed a significant increase after the intervention across participants (both autistic and typically developing). With respect to the possible effects of the FMS programs on social skills, the results are discordant. Some studies reported positive outcomes in the participants’ social abilities, as measured with the Social Skills Improvement System scales [6] and the Solitary Playground Observation of Peer Engagement scale [38]. Guest and colleagues [35] observed an improvement in the social skills domain in the Vineland Adaptive Behavior Scale-2 (VABS-2) but not in Social Skills Improvement System scores. This last result was in line with the study of Bremer et al. [32], although they reported no significant effects on the VABS-2 as well. Comparing fine motor and sensory integration interventions, Pfeiffer et al. [39] reported a significant decrease of mannerisms, as rated by parents with the Social Responsiveness Scale, in the sensory integration group. Lastly, the study that compared the outcomes of rhythm and robot interventions to those of an intervention with activities at a table [5] revealed no intergroup difference in imitation, as assessed with a program-specific test of imitation and praxis.

Interestingly, three of the reviewed studies also collected follow-up measures to evaluate the persistence of the interventions’ effects. Bremer and colleagues [32] performed a follow-up assessment six weeks after the end of intervention and observed a retention of motor improvements, as assessed with the Peabody Developmental Motor Scales-2, for one experimental group (the one had the longer treatment but less intense treatment) but not for the other one (the one that had the shorter but more intense treatment). Ketcheson et al. [38] confirmed a significant persistence in the improvements identified with the TGMD-2 scales four weeks after the end of treatment. Last, Guest and colleagues [35] had a follow-up eight weeks later that showed the retention of the improvements identified with TGMD-3.

## 4. Discussion

The purpose of the present narrative review was twofold. First, we aimed to provide an up-to-date overview of the findings from the studies that have used specific FMS interventions in autistic children over the past 10 years. Second, we intended to explore whether interventions that have specifically addressed FMS also included measures of autistic core characteristics and, in case, to consider the possible effect of FMS interventions on the sociocommunicative domain of autistic children. These issues have been representing emerging fields of interest for autism-related research through the last decade, given the systematic observations of motor deficits in autistic children (e.g., [3,4]) and following the hypothesis that early motor difficulties could significantly interfere with the development of sociocommunicative skills (e.g., [20]).

Although the literature is at a very early stage, some preliminary qualitative suggestions can be drawn from the reviewed studies. With respect to the first goal, the results of the present review are in line and extend those of Colombo-Dugovito and Block [28] and of Case and Yun [29], suggesting that specific FMS interventions could have a beneficial effect on the motor proficiency of autistic children. Indeed, nine of the ten reviewed studies reported significant improvements in a range of fine motor skills, gross motor skills, locomotor activity, and body coordination. Furthermore, the three studies which included follow-up evaluations of motor skills suggested a significant retention of FMS improvements four weeks [38], eight weeks [35], and even five months [32] after the end of the intervention. As highlighted by Colombo-Dugovito and Block [28], long-term maintenance of motor skills over time is a crucial question for FMS interventions, not sufficiently addressed at this stage. On the other hand, it is also important to acknowledge that only two works [5,34] among the reviewed studies were randomized clinical trials. Of these, the study of Felzer-Kim and Lynn Hauck [34] did not report statistically significant FMS improvements after intervention (although the authors reported, at this stage, only the partial interim results of a larger ongoing intervention). Thus, the limited evidence available from randomized clinical trials does not allow at this stage any definitive conclusions. More randomized clinical studies are warranted to replicate the predominantly positive results of the reviewed studies.

As for the systematic review of Colombo-Dagouvito and Block [28] and for the meta-analysis of Case and Yun [29], the studies included in the present work also differed in many methodological aspects, such as sample size, study duration, type and dosage of intervention. These variations limited the possibility to identify a direct link between a specific intervention and different behavioral outcomes. However, it is interesting to consider, also in light of the recent advances of autism-related research, that two of the reviewed studies made additional use of technologies, beyond the mediation of therapists, to implement the rehabilitation programs. In Srinivasan et al.’s study [5], children engaged with two robots: Nao—a humanoid robot used for imitation and synchrony-based games—and Rovio—a mobile robot that involved the children in walking games. The results showed greater improvements in the body-coordination composite score of the BOT-2 and in imitation in participants in the robot-mediated intervention compared to participants who received standard treatments. Edwards et al. [32] tested the effect of sports AVGs on FMS. The results revealed a lack of after-training improvements in the participants’ skill scores—as measured with quantitative measures—but a significant improvement in their self-perceptions of their motor skills. Beyond providing those preliminary—though discordant—results [5,32], those innovative studies showed the feasibility of an FMS intervention that includes technology to engage autistic children. The use of the technology—in particular, robots and virtual reality environments—in interventions for autistic individuals has rapidly increased in recent years [41]. Robots offer a unique type of interaction for autistic children, as they are highly attractive and more predictable than human partners [42,43,44]. Virtual reality can actively support learning by allowing autistic children to systematically manipulate sensory feedback and control the environment, which prevents social anxiety and distress [45,46,47]. Accordingly, more studies need to explore the feasibility and validity of technology-mediated FMS interventions. Indeed, as stated by Case and Yun [29], the current stage of literature seems to indicate that the use of technology and robots within motor environments is not yet fully understood.

With respect to the secondary aim of the present work, we became concerned about the lack of a clear relationship between the improvement in FMS reported in the reviewed studies and subsequent potential benefits in the socio-communicative domain. Only six out of ten studied included an assessment of social and communication skills [5,6,32,35,38,39]. This was concerning in consideration of the increasing evidence that early difficulties in basic motor skills could significantly hinder the development of sociocommunicative skills and could even have a role in the pathogenesis of the disorder [18,48,49]. Among these studies, five [6,32,35,38,39] reported some positive effects of motor training on social skills and mannerism, as assessed by parents, and on imitation skills and engagement in peer interaction, as rated through direct observation. One study did not find any positive effect [5]. Altogether, these findings are in line with the recent suggestions of Reinders and colleagues [18] which further documented the bidirectional relationship between social functioning and physical activity. However, considering the studies that described positive findings, we observed a number of inconsistencies between the scores of the different measures used to assess social and communicative skills. Thus, the available evidence about the potential effects of FMS interventions on core difficulties of ASD is still insufficient for drawing any conclusion. Beyond the scarcity of studies that measure sociocommunication outcome after FMS and small sample sizes of the reviewed ones, one reason for the results’ inconsistency could be directly related to the nature of the assessment process of ASD. Dimensions such as sociocommunication and adaptive functioning are complex to be quantified and the measures currently used for evaluating core features of ASD could be not sensitive enough to detect immediate changes after a specific, brief FMS training. From the ICF-CY perspective, the interconnection between motor skills and social abilities is a complex system rather than a linear causal relationship, as stated also by Reinders and colleagues [18]. This system can develop into a virtuous circle and it is important that future research on FMS intervention in ASD could find the best way to disentangle it. As recently suggested by Ruggeri and colleagues [50], it is crucial to identify the proper way to assess the impact of a body function improvement on the level of activity and participation of an individual as defined by ICF-CY. These dimensions are the actual indicators of the person’s adaptation and functioning in its own context. On the other hand, it could also be critical to individuate facilitators and barriers in the child’s environment, as factors to keep into consideration when quantifying activity and participation. Lastly, it is important that future studies include an assessment of the participants’ quality of life after treatment because it could be a reliable indicator of the intervention’s effectiveness [51].

The present review had some limitations. The goal of this work was to provide an up-to-date, narrative overview of recent findings on FMS interventions in autistic children. To do this, we limited our search to the literature published in the last 10 years that involved autistic participants between the ages of 3 and 12. In addition, even though we did not have any a priori language limitations, we considered only papers in English. Furthermore, we did not include single-case studies in consideration of the lack of generalizability of obtained effects. However, this decision could have significantly limited the present findings, because single-case designs formed a pivotal role in early motor skill development work. Given the exploratory nature of the present narrative review, we did not take into account the possible confounding role of co-occurring conditions or intellectual impairments. Some methodological aspects of the reviewed studies also limited our findings. Many interventions had small sample sizes, and only Felzer-Kim and Lynn Hauck [33] reported the effect size of their studies. Although small samples are frequent in daily clinical practice and the rehabilitation of autistic children, this could have limited the power of the studies considered by preventing the detection of even more significant behavioral outcomes. Further, only four studies indicated whether the pre- and posttreatment examiners were blind about the status of interventions. Remarkably, only three of ten interventions had follow-up assessments. Because the retention of learning skills for a significant period of time is a pivotal aspect of any treatment, future research on FMS interventions should systematically include a follow-up visit to evaluate the maintenance of possible behavioral benefits. Finally, future studies should consider exploring the validity of the video-modeling approach for developing FMS due to its demonstrated benefits for other skills such as sociocommunicative and daily living skills [52,53].

Despite these limitations, the present review preliminary suggests that FMS interventions could have some beneficial effects in autistic children, highlighting the importance of including motor skills training within the rehabilitation programs designed for children with this condition. This is particularly relevant because of the well-documented existence of motor impairments in autistic people. More work is needed to ascertain the possible impact of the positive motor outcomes on the participants’ social abilities.

## Figures and Tables

**Table 1:** Main characteristics of studies included in the present review.

Study	Location	Study Type	Participants (N, Groups)	Age Range (Mean ± SD)	Intervention Approach	Intervention Length	Setting	Outcome Measures	Results
**Bremer et al.** [6]	Canada	Multiple-method study with pre-/posttest	N = 5 (4 ASD, 1 autism-like characteristics) One group (1f/4m)	3–7 years (5.46 ± 1.49)	Fundamental motor skill intervention (FMSI): each session focused on teaching and strengthening of both locomotor and object control skills. Each week focused on teaching one core skill with previously learned skills integrated into the sessions.	12 weeks split into two blocks (each block: 45 min per day for 3 days per week) for a total of 36 sessions	Local elementary school	Test of Gross Motor Development-2 (TGMD-2); Social Skills Improvement System (SSIS); Motor-skill proficiency and social skills were assessed at 3 times: baseline, after Block 1 of the intervention, and after Block 2 of the intervention.	Improvements on the majority of the individual TGMD-2 items, 4 of the participants showed improvements in locomotor skills, furthermore 3 of the participants in object-control from Assessments 1–3. Improvements in SSIS on all items. The special education teacher noted the increase of motor skills and knowledge in the participants, the treatment program could be easily generalized and her own perception of her ability to teach physical education improved.
**Bremer et al.** [32]	Canada	Wait-list control experimental design with pre-/post-follow up test	N = 9 ASD 2 groups: EG N = 5 (5m); CG N = 4 (1f/3m)	4 years (EG = 4.30 ± 0.25; CG = 4.33 ± 0.22)	Fundamental motor skill intervention (FMSI): each session focused on teaching and strengthening of both locomotor and object control skills (they progressed in difficulty over the intervention period), while integrating previously learned skills into the review period and obstacle course.	Phase I: group 1 attended a 12-week FMS intervention for 1 h per week, while group 2 acted as a control. Phase II: group 2 attended a 6-week FMS intervention for 2 h each week (1 h per day on 2 separate days)	Local Children’s Treatment Centre	Peabody Developmental Motor Scales-2 (PDMS-2), considering the total motor quotient score provided as the primary outcome for the study; Vineland Adaptive Behavior Scales-2 (VABS-2); Social Skills Improvement System (SSIS); Behavioral video coding using Social Behavior Codes.	Intervention effectiveness: higher scores on object manipulation and total motor quotient PDMS-2 for EG compared to CG. No significant differences between two groups regarding adaptive behavior (VABS-2) and social skills (SSIS). Intensity effectiveness: time played a role on all PDMS-2 variables but not on adaptive behavior and social skills. No differences were found on outcomes between the two intervention intensities.
**Edwards et al.** [33]	Australia	Pre- and posttest experimental design	N = 30 (11 ASD, 8m/3f; 19 TD, 10m/9f)	6–10 years (ASD = 7.64 ± 1.12; TD = 7.89 ± 1.45)	Active Video Games (AVGs) like Kinect Sports Season 1, Kinect Sports Season 2, Sports Rivals and Kinect Adventures (TD group only).	ASD = 6 session (45/60 min each one, 3 times per week for 2 weeks) TD = 6 session (50 min each one, once a week for 6 weeks)	For ASD at home, for TD group at school during lunchtime	Test of Gross Motor Development-3 (TGMD-3) for OC (object control) skill improvement; Pictorial Scale of Perceived Movement Skill Competence (PMSC) for perceived OC skills.	There was no increase between pre- and postintervention for actual OC skill both in ASD and in TD group. Only in children with ASD, there was an increase of perception of skill.
**Felzer-Kim and Lynn Hauck** [34]	USA	RCT; pre-/posttest design	N = 14 ASD 2 groups: EG (1f/7m); CG (3f/3m)	4 years (EG = 4.5 ± 0.6; CG = 4.5 ± 0.6)	Fundamental motor skill intervention (FMSI): each session focused on training one of the 13 FMS. Each trial consisted of viewing a tablet-displayed video of the FMS, a picture task card and a verbal instruction. Then participants completed one trial of the skill, with physical prompt and reinforcement.	20 weeks split into two blocks (each block: 15 min per day for 4 days per week) for a total of 20 h	Campus of an ABA EIBI clinic	Test of Gross Motor Development-3 (TGMD-3) Anthropometrics (height; weight; BMI) Assessment at 4 times: baseline, at mid-intervention (10 weeks), post-intervention and follow up. Results presented here are only relative to the first two assessment points.	No significant interaction between time*group was found: 10 h of intervention did not alter FMS in this sample. Time*group interaction approached significance for ball skills and total but more time is necessary. Overall, locomotor skill improvement seems to be lower than ball skills and total skills.
**Guest et al.** [35]	Canada	Pre-/posttest quasi-experimental design with follow-up 8 weeks later.	15 ASD (13f)	8–11 (9.76 ± 1.00)	Special Olympics FUNdamentals program based on Long Term Athlete Development Model (LTAD) focused on locomotor skills and object control skills. Motor skills were incorporated into active group games and became difficult throughout the week.	Five full-day	School gymnasium	Test of Gross Motor Development-2 (TGMD-2) Time-stamped pedometer for physical activity Physical self-perceptions. The Children and Youth Physical Self-Perception Profile (CY-PSPP) Children’s Self-Perceptions of Adequacy in and Predilection for Physical Activity (CSAPPA) Social Skills Improvement System (SSIS) Adaptive Behavior Scales, 2nd edition (VABS-2)	Significant improvement after the intervention in Test of Gross Motor Development-2, physical self-perception in CY-PSPP, and social skills domain in VABS
**Hassani et al.** [36]	IRAN	Pre-/posttest design	30 ASD (10f/20m) 3 groups: EG-training 1 (4f/6m); EG-training2 (3f/8m); CG (3f/6m)	8–11 years (EG-training 1 = 9.10 ± 0.87; EG-training 2 = 8.55 ± 0.68; CG = 8.70 ± 0.70)	Training 1- SPARK: this protocol consisted of ten minutes for warm-up and 40 min for MS like balance skills, locomotor skills such Training 2—I Can Have a Physical Literacy (ICPL): this program focuses on MS like locomotor, balance, kicking, throwing and using of various tools such as visual cards	Sixteen indoor sessions, with two sessions of 60 min each per week performed after school.	Sport arena	Bruininks-Oseretsky Test of Motor Proficiency (BOT-2)	Both treatments incremented gross MS in comparison with the control group, with a major effect of ICPL group. Interestingly ICPL training also improved fine MS, unlike SPARK training.
**Henderson et al.** [37]	USA	Pre-/posttest design	37 ASD (35m, 2f)	5–12 (8.4 ± 2.06)	Physical education program targeting the six locomotor and six object control skills of the TGMD-2	40 min twice a week for six months, total: 40 classes	Gymnasium at a center for children with ASD	Test of Gross Motor Development-2 (TGMD-2)	Significant improvement after intervention for both gross motor skills and object control skills.
**Ketcheson et al.** [38]	USA	Pre-/post-follow-up test design	N = 20 ASD 2 groups: EG N = 11 (2f/9m); CT N = 9 (3f/6m)	4–6 years (EG = 4.87 ± 0.61; CG = 5.04 ± 0.61)	The intervention implies a weekly rotation between the Test of Gross Motor Development-2 (TGMD-2) subtests: locomotor skills and object control skills, using the eight components from the Classroom Pivotal Response Teaching (CPRT) manual as the framework for delivery of instruction.	4 h per day, 5 days per week for 8 weeks	Gymnasium and outdoor environment	Test of Gross Motor Development-2 (TGMD-2); Physical Activity monitor wearable; Playground Observation of Peer Engagement (POPE), only for EG.	Significant increase in motor proficiency in EG (locomotor skills, object control and gross quotient TGMD-2) compared with CG. A decrease in solitary scale in POPE was found. For all levels of PA, no significant group differences were observed. For joint engagement, parallel play and onlooking, no significant effects of time were found.
**Pfeiffer et al.** [39]	USA	Pilot study for subsequent randomized controlled trial; pre-/posttest design	N = 37 (21 ASD; 16 PDD-NOS) 2 groups: EG N = 20 (3f/17m); CG N = 17 (2f/15m)	6–12 years (EG = 8.3 ± 2.06; CG = 9.21 ± 2.06)	Sensory Integration (SI) based treatment: therapeutic activities characterized by enhanced sensation, especially tactile, vestibular, and proprioceptive, active participation, and adaptive interaction; Fine Motor (FM) intervention: focus on three main activity areas: (1) constructional, (2) drawing and writing, (3) FM crafts.	18 treatment interventions of 45 min each for 6 weeks	Three areas with appropriate equipment for SI; a separate room for FM.	Sensory Processing Measure (SPM); Social Responsiveness Scale (SRS); Quick Neurological Screening Test (QNST–II); Goal Attainment Scaling (GAS).	Decrease in mannerisms (a subscale of the SRS) in SI group; both groups showed significant improvement on the GAS, although the improvement was significantly greater in SI group. No significant differences between the two groups on sensory processing standardized scores (SPM), other subscales of SRS and the QNST–II.
**Srinivasan et al.** [5]	USA	RCT; pre-/posttest	N = 36 ASD 3 groups: EG1 N = 12 (2f/10m); EG2 N = 12 (1f/11m); CG N = 12 (1f/11m).	5–12 years (EG1 = 7.88 ± 2.56; EG2 = 7.52 ± 2.22; CG = 7.36 ± 2.02)	In the Rhythm and Robot group, gross motor skills including balance, bilateral coordination, imitation, interpersonal synchrony, and manual dexterity were trained whereas in the comparison group fine motor skills such as symmetrical and asymmetrical grips and pinches, coloring, drawing, cutting, and gluing were promoted. In all three groups, training enhanced social communication skills.	32 sessions (16 expert and 16 parent sessions) of 45 min each one over 8 weeks	Participants’ home	Bruininks-Oseretsky Test of Motor Proficiency (BOT-2); Training-Specific Test of Imitation/Praxis; Training-Specific Test of Interpersonal Synchrony.	Improvements in body coordination for both rhythm and robot group. Improvements on the fine manual control composite for the control group. Improvements on Imitation/praxis for all groups and on interpersonal synchrony for the two EGs. No improvement in fine motor performance for both rhythm and robot group. No improvement in the body coordination composite for the control group.

Note: EG = experimental group; CG = control group; ASD = autism spectrum disorder; m = male; f = female; RCT = randomized controlled trial; PDD-NOS = pervasive developmental disorder not otherwise specified; PA = physical activity; FMSI = fundamental motor skill intervention.
